# Generation of recombinant *Orf *virus using an enhanced green fluorescent protein reporter gene as a selectable marker

**DOI:** 10.1186/1746-6148-7-80

**Published:** 2011-12-22

**Authors:** Zhangyong Ning, Yongzheng Peng, Wenbo Hao, Chaohui Duan, Daniel L Rock, Shuhong Luo

**Affiliations:** 1 Department of Pathobiology, College of Veterinary Medicine, University of Illinois at Champaign-Urbana, 2001 S. Lincoln Avenue, Urbana, IL 61802, USA; 2 College of Veterinary Medicine, South China Agricultural University, Guangzhou 510642, People's Republic of China; 3 Department of Laboratory Medicine, Zhujiang Hospital, Southern Medical University, Guangzhou, 510280, People's Republic of China; 4 Institute of Antibody Engineering, School of Biotechnology, Southern Medical University, 1838 N. Guangzhou Avenue,Guangzhou, 510515, People's Republic of China; 5 Laboratory of Clinical Immunology, the Sun Yat-Sen Memorial hospital, Sun Yat-Sen University, Guangzhou 510120, Guangdong, People's Republic of China

## Abstract

**Background:**

Reporter genes are often used as a selectable marker for generation of recombinant viruses in order to investigate the mechanism of pathogenesis and to obtain candidate vaccine viruses. Routine selection of the recombinant parapoxvirus is time-consuming and labor intensive. Therefore, developing a novel method for selection is critical.

**Results:**

In this study, we developed a rapid method to generate recombinant Orf viruses (ORFV) based on the enhanced green fluorescent protein (EGFP) reporter gene as a selectable marker. The coding sequence of *EGFP *gene was amplified from pEGFP-N1 vector and subcloned into the pZIPPY-neo/gus plasmid under the control of the early-late vaccinia virus (VACV) VV7.5 promoter and flanked by two multiple cloning sites (MCS) to generate a novel transfer vector pSPV-EGFP. Using the pSPV-EGFP, two recombination cassettes pSPV-113LF-EGFP-113RF and pSPV-116LF-EGFP-116RF were constructed by cloning the flanking regions of the ORFV113 and ORFV116 and inserted into two MCS flanking the EGFP gene. Using this novel system, two single gene deletion mutants OV-IA82Δ113 and OV-IA82Δ116 were successfully generated.

**Conclusions:**

This approach shortens the time needed to generate recombinant ORFVs (rORFVs). Thus, the pSPV-EGFP vector provides a direct, fast, and convenient way to manipulate the recombinant viruses, indicating that it is highly suited for its designed purpose.

## Background

Orf virus (ORFV), the prototypic member of the Parapoxviridae, is the cause of a papular demertitis in sheep and goats known as contagious ecthyma [1]. ORFV has been used in veterinary medicine as a preventive and therapeutic immunomodulatory agent. Live or inactivated ORFV preparations exhibit dose-dependent immunomodulatory effects when administered to multiple animal species including cattle, horses, cats and dogs. Significant therapeutic efficacy of ORFV preparations in preventing or treating stress-associated and other infectious disease conditions has been well documented [1-4]. However, ORFV functions (genes/proteins/mechanisms of action) associated with modulation and manipulation of host immune responses are still poorly understood.

Previously we identified 16 novel, mostly terminally located ORFV genes with putative virulence and host range functions [5], hypothesizing that these proteins perform novel but undescribed immunomodulatory functions in the host. To investigate the role of these genes during virus infection, one approach to studying uncharacterized genes is to create mutations and/or deletions of specific ORFV genes to disrupt their function. This can be achieved by using transfer vectors designed to insert into site-specific locations of the viral genome via homologous recombination [6]. However, the experimental procedures to generate and isolate rORFVs are adapted from standard protocols used in generation of the vaccinia virus. This protocol is labor intensive and time consuming [7,8].

The Green Fluorescent Protein (GFP) was discovered by Shimomura et al as a companion protein to aequorin [9], the famous chemiluminescent protein from *Aequorea *jellyfish which exhibits bright green fluorescence when exposed to blue light [10,11]. In cellular and molecular biology, the *GFP *gene is frequently used as a reporter of expression [12]. Lately, the Enhanced Green Fluorescent Protein (EGFP) was developed and has been introduced and expressed in many bacteria [13], yeast and other fungi [14], fish [15], plants [16], flies [17] and mammalian cells [18] including human. In recent years, recombinant GFP/EGFP expression coupled with flow cytometry to produce an individual cell-based readout with increased sensitivity has been widely utilized to generate recombinant vaccinia viruses to study virus tropism and a high-throughput vaccinia virus neutralization assay [19-22].

In the present study, to shorten the time required to generate rORFVs, we took advantage of *EGFP *reporter gene as a selection marker to isolate rORFVs. The novel transfer vector pSPV-EGFP was constructed to express EGFP in rORFVs. Using this novel system, two single-gene deletion mutants OV-IA82Δ113 and OV-IA82Δ116 were successfully generated. This new approach allowed us to generate the desired rORFVs in less than 15 days. Compared to the neo/gusA selection, EGFP selection provides a direct, fast and convenient way to construct recombinant viruses, indicating the usefulness of the pSPV-EGFP vector.

## Results

### Generation of rORFVs using neo/gusA selection

To generate rORFVs, the convenient and efficient methods for identifying recombinants are necessary. In recent years, to improve the efficiency of recombinant isolation, the combination of drug selection and identifiable color detection provides an advantage over previous methods in the poxvirus field [7]. To investigate the functions of these novel genes encoded by ORFV, construction of gene-deleted mutant viruses is a first step. In previous studies [23-25], the pZIPPY-neo/gus vector containing the *neo/gusA *cassette was initially used to construct recombinant cassettes based on the flanking regions of ORFV002, ORFV012, ORFV024, ORFV113, ORFV116, ORFV120 and ORFV121. The preliminary results were summarized in Table 1. Using this strategy, four single gene deleted-mutant recombinants OV-IA82Δ002 [23], OV-IA82Δ024 [24], OV-IA82Δ120 (unpublished data), OV-IA82Δ121 [25] were finally generated and isolated through 17 and 20 rounds of plaque purification (each round takes at least 4 or 5 days to pick blue plaques, Table 1). However, OV-IA82Δ012, OV-IA82Δ113, and OV-IA82Δ116 are still contaminated with the parent virus after more then 20 rounds of plaque purification (Table 1). This protocol is labor intensive and time consuming, requiring at least 3 or 4 months to isolate and purify recombinants. A new strategy to generate rORFVs is critical.

**Table 1:** Comparison of generating rORFVs using the pSPV-EGFP and pZIPPY-neo/gus vectors

OV gene	Rounds of plaque assay		Time to obtain the puried rOVs (days)	
	
	pSPV-EGFP	pZIPPY-neo/gus	pSPV-EGFP	pZIPPY-neo/gus
ORFV002	-^a^	16	-	80[23]^b^
ORFV012	-	20	-	>100^C^
ORFV024	-	19	-	95[24]
ORFV113	4	20	12	>100^C^
ORFV116	5	20	14	>100^C^
ORFV120	-	17	-	85
ORFV121	-	18	-	90[25]

^a ^Not tested; ^b ^Reference; ^c ^After 20 rounds of plaque assay, still contaminated with wild type virus.

### Construction of recombinant cassettes with the novel transfer vector pSPV-EGFP

**Figure 1:**
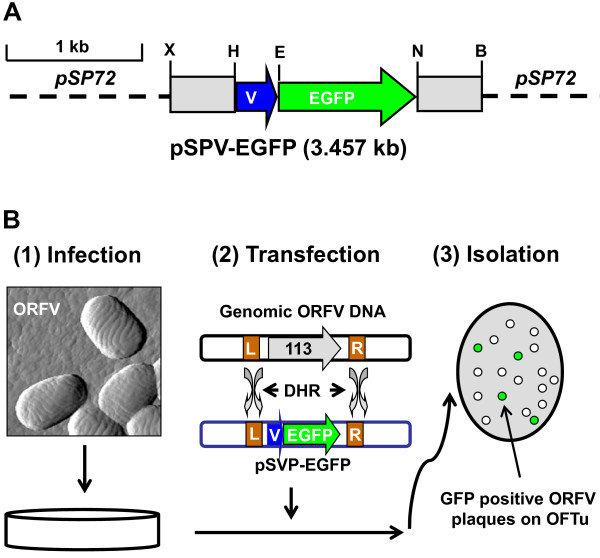
**Infection/transfection scheme for generation of recombinant ORFV**. **A**. Construction of the recombinant transfer vector pSPV-EGFP. A cassette of selectable markers of *E.coli neo *and *gusA *genes in pZIPPY-neo/gus vector was replace by the *EGFP *reporter gene amplified from pEGFP-N1 vector (Clontech, CA) to generate the recombinant vector pSPV-EGFP. **B**. Generation of recombinant ORFV. OFTU Cells are infected with OV-IA82 and transfected with the transfer vector pSPV-113LF-EGFP-113RF. The resultant virus mixture is then plated on OFTu cells to eliminate OV-IA82 and the desired viruses were isolated. MCS: Multiple cloning sites. B: BglII; E: EcoRI; H: HindIII; N: NotI; and X: XhoI. V: vaccinia virus (strain WR) VV early/late protomer VVp7.5. L: Up stream of ORFV113 un-transcription region; R: Down stream of ORFV113 un-transcription region. DHR: double homologous recombination.

To shorten the time needed to generate rORFVs, the novel transfer vector pSPV-EGFP (Figure 1A) was constructed using the *EGFP *reporter gene to replace the *neo/gusA *cassette [7]. To compare the efficiency of plaque purification between pZIPPY-neo/gusA [7] and pSPV-EGFP, two ORFV proteins, ORFV113 and ORFV116, were used for this study. Two transfer vectors, pSPV-113LF-EGFP-113RF and pSPV-116LF-EGFP-116RF, were constructed using pSPV-EGFP. These cassettes encode EGFP under the control of the early-late VV 7.5 promoter, which allows for isolation and purification of recombinant viruses by using fluorescent signals under a fluorescent microscope. This novel experimental strategy streamlines the procedure. The experimental steps in this new strategy include: (i) generating the OV-IA82Δ113 and OV-IA82Δ116 after transfection of the pSPV-113LF-EGFP-113RF and pSPV-116LF-EGFP-116RF vectors into ovine fetal turbinate (OFTu) cells previously inoculated with OV-IA82; (ii) visual monitoring of infected cultures by fluorescent microscopy to assess the level of rORFV infection; (iii) determine the optimal time (usually 24 hours during the second round of limited dilution) to harvest cells for plaque assay (Figure 1B).

### Generation of OV-IA82Δ113 and OV-IA82Δ116 using EGFP selection

**Figure 2:**
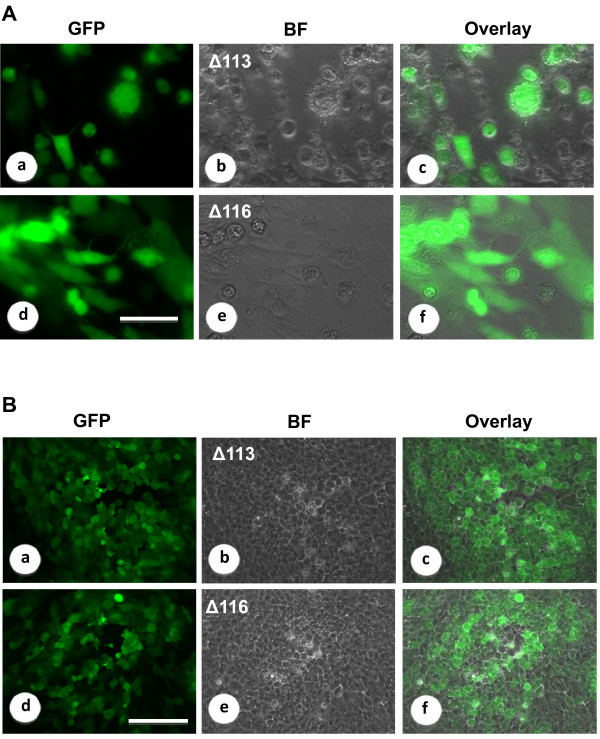
**Fluorescent microscopy showing the cytopathic effects (CPE) of OFTu cells infected with OV-IA82Δ113 and OV-IA82Δ116 by using limited dilution (A) and plaque purification (B)**. **A**. Limited dilution showed OFTu cells infected with OV-IA82Δ113 and OV-IA82Δ116 after 24 h pi. The strong fluorescent signal was observed in OFTu cells infected with both OV-IA82Δ113 and OV-IA82Δ116. (b) and (e) show the same fields as in (a) and (d) by bright-field microscopy. (c) and (f), overlay of (a) and (b) or (d) and (e). Cells were visualized in a Leica SP2 microscope in 63 × magnification. Bar, 10 μm. **B**. Plaque purification showed the plaques with strong fluorescent signal in OFTu cells infected with OV-IA82Δ113 (a, b, c) and OV-IA82Δ116 (d, e, f) at 36 h pi during the second round of plaque purification. (b) and (e) show the same fields as in (a) and (d) by bright-field microscopy. (c) and (f), overlay of (a) and (b) or (d) and (e). Cells were visualized in a Leica DMI4000B inverted microscope in 10× magnification. Bar, 20 μm.

When the pSPV-113LF-EGFP-113RF and pSPV-116LF-EGFP-116RF vectors were transfected into OFTu cells that had been previously exposed to OV-IA82 with 1.0 multiplicity of infection (MOI), spots of brightly EGFP-positive cells appeared within 6 h, and strong fluorescent signal was observed under fluorescent microscope after 24 h. Cytopathic effect (CPE) of OFTU cells infected with OV-IA82Δ113 or OV-IA82Δ116 (Figure 2A) was observed after 24 h during the second round of limited dilution, and abundant EGFP expression was observed in infected OFTu cells. EGFP-positive cells in 96-well plates with dilutions higher than 1:10, 000 were harvested for further plaque purification. After two rounds of limited dilutions, three different individual recombinant clones for each protein were selected to run 2 or 3 times plaque assays to remove parent virus contamination. Plaques with a fluorescent signal can be identified in OFTu cells after 12 h pi, and individual green plaques were picked under fluorescent microscope at 36 h pi (Figure 2B). This demonstrates that EGFP selection substantially shortens the time than conventional protocols such as *neo/gusA *selection.

### Identification of OV-IA82Δ113 and OV-IA82Δ116

**Figure 3:**
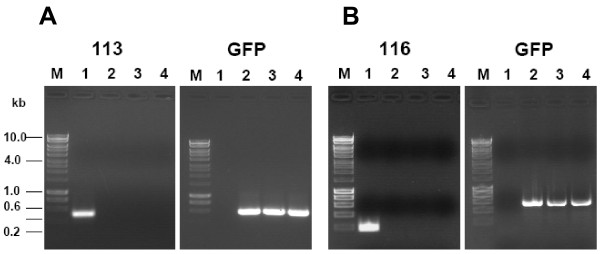
**PCR detection of *EGFP *gene and *ORFV113 *or *ORFV116 *in OV-IA82Δ113 and OV-IA82Δ116**. Genomic DNA was isolated from OV-IA82 and OV-IA82Δ113 (A) or OV-IA82Δ116 (B). PCR was preformed to confirm the absence of *ORFV113 *(A) and *ORFV116 *(B) and the presence of the *EGFP *reporter gene sequences in OV-IA82Δ113 and OV-IA82Δ116 genomes. M: molecular marker (HyperLadder I, Bioline); Lane 1: OV-IA82; Lane 2 to 4: three different recombinants of OV-IA82Δ113 (A) and OV-IA82Δ116 (B).

To determine the purity of OV-IA82Δ113 and OV-IA82Δ116, PCR was conducted using primers designed from internal coding regions of ORFV113 and ORFV116 or EGFP (Table 2). The results demonstrated that the *EGFP *gene was amplified from three different clones of OV-IA82Δ113 (Figure 3A) and OV-IA82Δ116 (Figure 3B), but not from OV-IA82 or mock infected cells. In contrast, the *ORFV113 *and *ORFV116 *genes were amplified from OV-IA82 infected cells only (Figure 3).

**Table 2:** Oligonucleotide primers and restriction enzymes

Primer	Sequence	Restriction enzyme*
GFPFw1	5'-AACTTAGAATTCGCCACCATGGTGAGCAAGGGCGA-3'	EcoR I
GFPRv1	5'-ATCAATGCGGCCGCTTACTTGTACAGCTCGTCCA-3'	Not I
GFPintrFw2	5'-GACGTAAACGGCCACAAGTT-3'	
GFPintrRv2	5'-ACTGGGTGCTCAGGTAGTGG-3'	
GFPseqFw3	5'-CGACCACTACCAGCAGAACA-3'	
GFPseqRv3	5'-AAGTCGTGCTGCTTCATGTG-3'	
113LFFw1	5'-AGGCCTCTAAGCTTCAGGTTCCGGCTTCAGATGCGCGT-3'	Hind III
113LFRv1	5'-ATTCGCGTCGACCACCAACACTTCCATTGTTGCGGC-3'	Sal I
113RFFw2	5'-TCTTATGCGGCCGCGAGCCGCCGATGCAGATCGAGGTA-3'	Not I
113RFRv2	5'-ATTCGCAGATCTTCGATCGCCAGTGCGCGGCGCATG-3'	Bgl II
113intrFw3	5'-CGCCGTAATATGCTTAACCGGAGC-3'	
113intrRv3	5'-CGGACCGTGTTGGTCGTTGGGTCT-3'	
113seqFw4	5'-TTAGCTTCCTTGTTTTTATC-3'	
113seqRv4	5'-GTCCTTCGGGTCAGAGTCC-3'	
116LFFw1	5'-GCCTCTACTAGTAGGAAGTGGCCTCGCCGACCACGA-3'	Spe I
116LFRv1	5'-ATTCGCGTCGACGTGGATGTCTCTAAGGTTCAATAC-3'	Sal I
116RFFw2	5'-TCTTATGCGGCCGCCTACCACTGGTACCAGCACCTCCT-3'	Not I
116RFRv2	5'-ATTCGCAGATCTGGCGCTACAGGCGTCCTGCAGGAA-3'	Bgl II
116intrFw3	5'-GAACAACACGTCAACCGATG-3'	
116intrRv3	5'-AGGTGTGGGTTGACTTCCAG-3'	
116seqFw4	5'-GTCGAGCAGATGTTCATGGA-3'	
116seqRv4	5'-ATGCTGCACTTCCTGGAGAT-3'	
001intrFw1	5'-CTCGGTGACCTGCCTGAC-3'	
001intrRv1	5'-CTCGCGCACGTCGTAGAT-3'	

* Restriction enzyme sites are underlined in prime sequences

**Figure 4:**
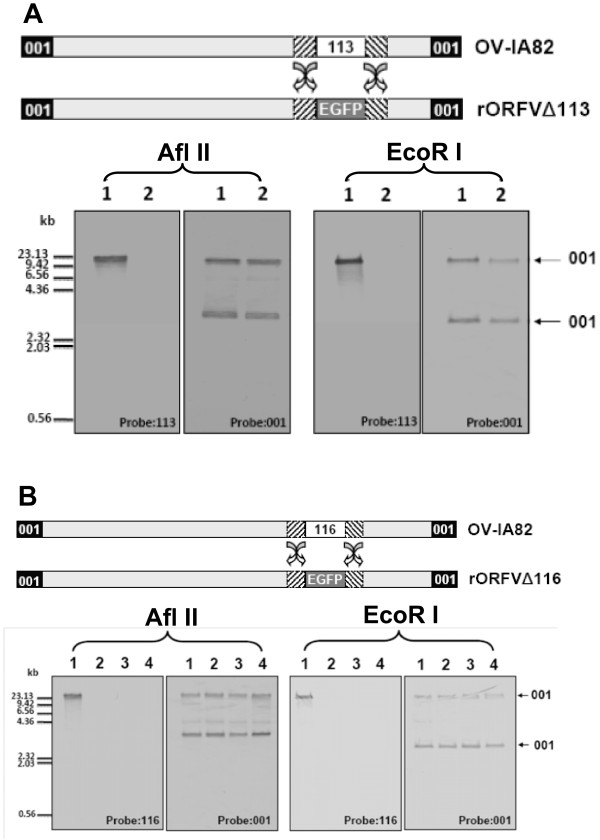
**Characterization of mutants of OV-IA82Δ113 and OV-IA82Δ116 by Southern blotting**. **A**. OV-IA82Δ113. Upper panel is a schematic of OV-IA82 genome before and after removal of the *113 *gene using the deletion vector, pSPV-EGFP by double homologous recombination to generate gene-deletion mutant OV-IA82Δ113. Lower panel shows Southern blot analysis. Genomic DNA was isolated from OV-IA82 (lane 1) and OV-IA82Δ113 (lane 2) and digested with restriction enzyme AflII or EcoRI respectively. The *113 *internal probe was unable to detect the *113 *gene in the recombinant OV-IA82Δ113 genome, indicating that the *113 *gene was completely removed from the *113 *locus of the genome. The *001 *probe detected both end of the *001 *loci in both OV-IA82 and OV-IA82Δ113 genomes. **B**. OV-IA82Δ116. Upper panel A is a schematic of OV-IA82 genome before and after removal of the *116 *gene using the deletion vector, pSPV-EGFP by double homologous recombination to generate gene-deletion mutant OV-IA82Δ116. Lower panel shows the *116 *gene, which was completely removed from the *116 *locus of the OV-IA82 genome by Southern blot analysis. The *116 *internal probe was unable to detect the *116 *gene in the OV-IA82Δ116 genome. The *001 *probe detected both end of sequences in OV-IA82 (lane: 1) and three different clones of OV-IA82Δ116 (lanes: 2 to 4).

To verify *ORFV113 *and *ORFV116 *genes were successfully deleted from OV-IA82 via the transfer vector pSPV-EGFP and the *EGFP *gene was incorporated into the OV-IA82 genome by homologous recombination; Southern blot analysis was performed on genomic DNA isolated from OV-IA82, OV-IA82Δ113 and OV-IA82Δ116. The *ORFV113 *probe detected the *ORFV113 *gene in OV-IA82 (Figure 4A lower panel, lane 1) but not in OV-IA82Δ113 (Figure 4A lower panel, lane 2). The *ORFV001 *gene is the only double copy located at the left and right ends of the viral genome. The *ORFV001 *probe detected the same pattern in both OV-IA82 and OV-IA82Δ113, indicating that the deletion of *ORFV113 *does not affect the left and right ends of the viral genome. The *EGFP *probe detected the *EGFP *gene in OV-IA82Δ113 but not in OV-IA82 (data not shown). Similar results were obtained when the *ORFV116 *probe was applied (Figure 4B). Sequencing of left and right flanking regions of the deleted gene, which were involved in the recombination, confirmed the integrity of parental virus sequences in the mutant virus (data not shown).

### Deletion of *ORFV113 *and *ORFV116 *do not affect ORFV replication *in vitro*

**Figure 5:**
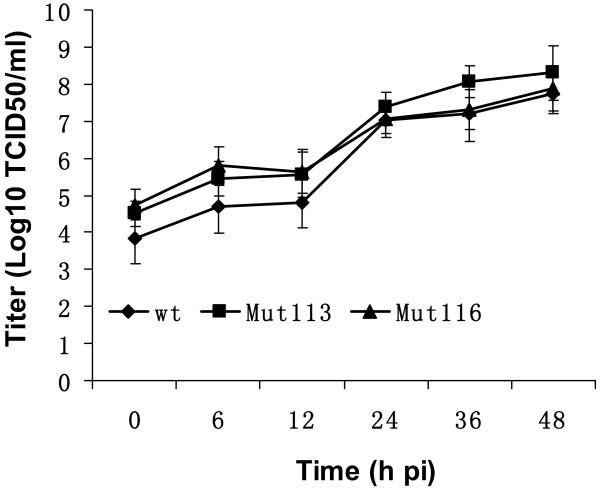
**Replication characteristics of OV-IA82Δ113 and OV-IA82Δ116 in OFTu cells**. One step growth curves were performed in OFTu cells. Statistics showed that there was no significant difference between wild type and mutant viruses (P > 0.05). Data represent the averages of the results of three independent experiments. Error bars show standard deviations. Diamond: OV-IA82; Square: OV-IA82 Δ113; Triangle: OV-IA82Δ116.

To investigate the infectivity of the gene-deleted mutant viruses, a one-step growth curve was conducted. Replication properties of OV-IA82Δ113, OV-IA82Δ116, and OV-IA82 were compared after infection of OFTu cells. No significant differences in growth were detected between the mutant and wild type viruses (P > 0.05) in OFTu cells, indicating that ORFV113 and ORFV116 are not essential for ORFV replication in OFTu cells (Figure 5). The data also demonstrates that expression of EGFP in OV-IA82Δ113 and OV-IA82Δ116 does not affect ORFV growth and replication *in vitro*. Also shown in our previous report [23], the exogenous expression of EGFP did not affect the viral phenotype and the biological function in ORFV002 revertant virus (OV-IA82Rv002GFP) which was constructed by using the pSPV-EGFP system. Taken together, the pSPV-EGFP vector could be widely used for recombination and foreign gene expression studies.

## Discussion

We have shown that generating rORFVs is easy and efficient using the EGFP reporter gene and is faster than conventional neo/gusA methods. Using this novel system, two single gene deletion mutants OV-IA82Δ113 and OV-IA82Δ116 were successfully generated. Additional gene deletion mutants such as ORFV001, ORFV005, ORFV012 and ORFV124 are under way. The advantages of EGFP fluorescent selection compared to conventional strategies are as follows: (i) easy to construct recombinant cassettes using the pSPV-EGFP; (ii) visual monitoring of infected cells by fluorescent microscopy to assess the level of rORFV infection; (iii) easy to determine the optimal time to harvest cells; (iv) streamlining the procedures and shortening the time needed to isolate and purify the plaques (less than 15 days), compared to 3 or 4 months using *neo/gusA *method (Table 1); (v) picking plaques directly under fluorescent microscope or by fluorescence activated cell sorting (FACS) within 24 h pi, no antibiotics and substrates needed; (vi) using the pSPV-EGFP plus the pZIPPY-neo/gus allows to make a double gene deletion mutants for multiple copy genes or the same cluster of gene family. Moreover, EGFP expression in infected cells provides a reliable, straightforward methodology to maintain viral stocks free from contamination with spontaneous ORFV revertants by the rapid selection marker of GFP-positive cells. Furthermore, the growth curve demonstrate that EGFP expression in recombinant viruses does not affect the virus replication (Figure 5) and the luciferase assay results of our previous reported OV-IA82Rv002GFP [23] also showed that the EGFP expression does not affect the virus biological functions. Previous studies have shown that recombinant vaccinia virus-mediated EGFP expression has been used to monitor infection *in vitro *[19] or *in vivo *[26]. Our EGFP-based vector construct may represent a practical tool to study ORFV infection and pathogenesis in natural hosts.

## Conclusions

In this study, the pSPV-EGFP vector, which contains two MCSs for insertion ORFV genes or foreign genes, provides a reliable, straightforward methodology for the advanced studies of ORFV replication, host range, as well as foreign gene expression. Using this new approach to generate and isolate rORFVs based on the EGFP selection marker remarkably reduces the time for recombinant virus isolation and purification. In addition, EGFP localization will give the opportunity to study host-virus interaction at the cellular level *in vivo*. Taken together, the novel transfer vector pSPV-EGFP provides an efficient and convenient way for gene deletion from OV-IA82 genome to study the novel gene functions and may also be used to acquire gene-deleted mutants from other viruses.

## Methods

### Cells and viruses

Primary ovine fetal turbinate (OFTu) cells were kindly provided by Dr. Howard D. Lehmkuhl (USDA) and were maintained in minimal essential medium (MEM) supplemented with 10% fetal bovine serum (FBS), 100 μg/ml streptomycin,100 U/ml penicillin, 50 μg/ml gentamicin and 2 mM L-glutamine.

ORFV IA82 (OV-IA82) strain was isolated from lamb nasal secretion during an orf outbreak at the Iowa Ram Test station in 1982, and has been fully sequenced [5]. Low passage OV-IA82, which is fully virulent, was used to construct deletion mutants OV-IA82Δ113 (Figure 4A) and OV-IA-82Δ116 (Figure 4B) and was used here in all procedures involving infections with wild type virus, PCR amplification and cloning of viral genes. The virus was propagated in OFTu cells and was purified by sucrose gradient ultra-centrifugation before storage at -80°C for use.

### Construction of pSPV-EGFP

To obtain the *EGFP *gene sequence, two primers were designed, sense-primer GFPFw1 and antisense-primer GFPRv1. An EcoR I site was added to the sense-primer and Not I to the antisense-primer (Table 2). Thermostable high fidelity DNA polymerase and dNTPs were obtained from Invitrogen (Carlsbad, CA, USA). Polymerase chain reaction (PCR) was performed in 50 μl thin-wall polypropylene tubes (Denville Scientific, NJ, USA) using a PTC-200 DNA Engine thermal cycler (Bio-Rad, Shelton, CA, USA) under the following conditions: denaturation at 96°C for 2 min followed by 30 cycles of denaturation at 94°C for 45 s, annealing at 54°C for 45 s, elongation at 72°C for 1 min, and a final extension at 72°C for 7 min. PCR products were visualized in 1% agarose gel with ethidium bromide staining, cut out, and purified using a Qiagen gel extraction kit (Qiagen, Valencia, CA, USA). The products were then cloned into vector pZIPPY-neo/gus [7], which had been linearized with EcoRI and NotI (New England Biolabs, Ipswich, MA, USA) to generate the novel transfer vector pSPV-EGFP (Figure 1A). The plasmid was propagated in *Escherichia coli *strain Top10 (Invitrogen, Carlsbad, CA, USA). DNA sequencing of the pSPV-EGFP was done to ensure that the correct construct had been obtained. The complete DNA sequence of the pSPV-EGFP was deposited in GenBank under the accession number: GU062789.

### Construction of recombinant cassettes

DNA preparations and manipulations were performed using standard methods as described by Sambrook et al. [27] or followed instructions from manufactures. Two recombination cassettes were constructed by PCR amplifying *ORFV113 *and *ORFV116 *left and right flanking regions from OV-IA82 genome using primers listed in Table 2. The PCR products were cloned into the pSPV-EGFP MCSs to generate **pSPV-113LF-EGFP-113RF and pSPV-116LF-EGFP-116RF**. In addition, to compare the efficiency between the pSVP-EGFP and pZIPPY-neo/gus, seven recombinant cassettes were also constructed using pZIPPY-neo/gus vector. The *ORFV002*, *ORFV012*, *ORFV024*, *ORFV113*, *ORFV116*, *ORFV120 *and *ORFV121 *left and right flanking regions from OV-IA82 genome were amplified and cloned into the pZIPPY-neo/gus MCSs, flanking the neomycin resistance (*neo*) and β-glucuronidase reporter (*gus*) genes under control of the VACV VV7.5 and modified H5 promoters, respectively [7]. The resulting recombinant vectors were named pZNG-ORFV002 [23], pZNG-ORFV012, pZNG-ORFV024 [24], pZNG-ORFV113, pZNG-ORFV116, pZNG-ORFV120, pZNG-ORFV121 [25]. All sub-cloning procedures were confirmed by using restriction enzymes and DNA sequence analysis.

### Generation of recombinant viruses

OV-IA82Δ113 and OV-IA82Δ116 viruses were constructed by infecting OFTu cells (in T25 flasks) at a multiplicity of infection (MOI) of 1.0 with wild type OV-IA 82 for 3 hours and subsequently transfecting the cells with 10 μg of pSVP-113LF-EGFP-113RF and pSVP-116LF-EGFP-116RF transfer vectors by standard *in vivo *recombination protocols [28,29]. Transfections were carried out using Lipofectamine 2000 (Invitrogen, Carlsbad, CA, USA) according to the manufacturer's instructions.

Viruses were harvested 48 h pi by scraping infected/transfected OFTu cells into sterile 15 ml conical tubes. The cell suspensions were vortexed, frozen/thawed 3 times, and then centrifuged at 1000 rpm, for 10 min at 4°C (Eppendorf Centrifuge 5810R, 15 amp version, Hamburg, Germany). The supernatant (viruses) were transferred into 2 ml cryogenic vials (Corning, NY, USA) and stored at -80°C for future use.

In order to select and purify recombinant viruses, limited dilution and plaque assays were performed. 3 ×10^4 ^of OFTu cells per well were seeded into 96-well plates one day before infection. On the second day, the cells were infected with serial 10-fold dilutions of viruses from 10^-1 ^to 10^-11 ^(900 μl 1 × MEM + 100 μl of virus). The cells were examined under a fluorescent microscope (Leica DMI4000B inverted microscope) 12 h after inoculation. The cells exhibiting fluorescent signal with dilutions higher than 1:10, 000 were collected for the second round of limited dilution. Typically, after 24 h pi, strong green fluorescent signal was observed in 96-well plates. The cell suspensions were harvested and frozen/thawed as above. The supernatant (viruses) was stored at -80°C for use.

After 2 or 3 rounds of limited dilution, plaque assays were carried out for further purification. 7 × 10^5 ^of OFTu cells per well were passed into 6-well plates one day before infection. On the next day, cells were infected with serial dilutions of viruses from 10^-1 ^to 10^-6^. The viruses were allowed to absorb to the cells for 1 h at 37°C, in 5% CO_2 _incubator. The medium was removed and then the cells were overlaid with 3 ml of MEM containing 5% of FBS and 0.5% low melting point agarose (Sea Kem^® ^GTG^®^, Lonza, Rockland, ME, USA). Plaques with a GFP signal were visualized and picked 24 or 36 h pi. A minimum of 2 or 3 plaques were picked for further plaque purification. The last plaque purified recombinant viruses were expanded in 35 × 10 mm dishes; genomic DNA was extracted using QIAamp DNA blood kit (QIAGEN, Germany). PCR was performed to screen for the wild type virus contamination using internal primers from *ORFV113 *and *ORFV116 *coding regions (Table 2). PCR conditions were the same as described above except that the annealing temperature was based on the Tm values of each primer. Southern blotting was further carried out to confirm that double homologous recombination had occurred.

For comparison studies, OV-IAΔ002, ORFVΔ012, OV-IA-82Δ024, OV-IA82Δ113, OV-IA82Δ116, OV-IA82Δ120 and OV-IA82Δ121 were also constructed using *neo/gus *selection following the procedures as described above. The only difference is that those recombinant viruses produced blue plaques (*gusA *activity) in the presence of X-gluc (Clontech, Palo Alto, CA, USA). Blue plaques were picked at day 4 or day 5 pi.

### Southern blots

Genomic DNA was extracted from OV-IA82, OV-IA82Δ113 and OV-IA82Δ116 viruses, respectively. One microgram of viral DNA was digested with AflII or EcoRI (New England Biolabs, Inc.), electrophoresed via a 1.0% agarose gel and transferred via capillary action to a nylon membrane (Bio-Rad, CA) using standard protocols [30]. The membranes were hybridized with specific digoxin (Roche, Mannheim, Germany) labeled probes, which were generated specifically for the *ORFV113*, *ORFV116*, *ORFV001 *and *EGFP *genes. The probes for ORFV113, ORFV116, EGFP and ORFV001 were amplified by using internal primers (Table 2), same primers applied for PCR detection as described above. Amplicons were purified and labeled with digoxin followed the manufacture's instruction (Roche, Mannheim, Germany). To determine the ends of recombinant viruses, the same blots were stripped with N, N-dimethylformamide (Sigma, St. Louis, MO, USA) at 50-60°C for 1 h, until color had been removed completely. During the stripping process, the solution needed to be changed every 20 min. The stripped membrane was incubated in stripping buffer (0.2 M NaOH and 0.1% sodium dodecyl sulfate) for 30 min at 37°C, and re-hybridized with a specific digoxin labeled probe that were generated toward the *ORFV001 *gene cassette. The NBT/BCIP Detection kit (Roche, Mannheim, Germany) for nonradioactive color development was used for hybridization analysis of Southern blots according to the manufacture's instructions.

### Sequencing analysis

To determine homologous recombination, three plaques, which were confirmed by PCR detection using *ORFV113 *and *ORFV116 *internal primers (113intrFw3 and 113intrRv3 or 116intrFw3 and 116intrRv3, table 2) were applied for sequencing analysis. Two pairs of primers (Table 2), 113seqFw4 and GFPseqRv3; GFPseqFw3 and 113seqRv4 or 116seqFw4 and GFPseqRv3; GFPFw3 and 116seqRv4, were utilized to amplify the regions involved in recombination. PCR conditions were the same as described above except that the annealing temperature was employed based on the Tm values of each primer. The PCR products were cloned into pCR2.1 TA cloning plasmid (Invitrogen, CA) for sequencing analysis using an Applied Biosystems PRISM 3730 automated DNA sequencer (Applied Biosystems, Foster City, CA, USA).

### Virus expansion, titration, and growth curve

Based on the Southern blot and sequence analysis, one clone of OV-IA82Δ113 and OV-IA82Δ116 were used for expansion. Confluent monolayers of OFTu cells in four T150 cm^2 ^flasks were infected at MOI of 1. The viruses were harvested when all cells were rounded but still attached (about 3 or 4 days pi). The cell suspensions were prepared as above. The supernatants were dispensed into 2 ml cryogenic vials (1 ml/vial) and stored at -80°C for future use.

96-well plates of 90% confluent monolayers OFTu cells were prepared as above. Rows of cells were infected with serial 10-fold dilutions of wild type and mutant viruses of 10^-1 ^to 10^-9^, with one row for control (no virus). One column was used for one dilution. CPE was read at 2 d pi, and the final read was carried out at 7 d pi. The median tissue culture infected dose (TCID_50_) per ml was calculated using a spreadsheet.

One step growth curves were conducted as our pervious reports [23-25]. OFTu cells were prepared in 35 × 10 mm dishes (7 × 10^5 ^cells/dish) one day before the experiment. The cells were counted on the second day and were infected at a MOI of 10 with wild type and mutant virus. Virus was harvested at 0, 6, 12, 24, 36, 48 h pi and titrated. The growth curves were plotted as titer (log_10 _TCID_50_/ml) versus time course (h) pi. Data are presented as the mean ± standard deviation from three independent experiments. *P *values were determined by using the unpaired two-tailed Student's t-test. Statistical significance was set at the 95% confidence level.

## Authors' contributions

SL, ZN and DR participated in design of the study. CD and WH constructed the plasmid pSPV-EGFP. ZN, YP and WH prepared the recombinant virus and get the grow curve and Southern blot analysis. ZN, SL and DR analyzed the data and wrote the manuscript. All authors read and approved the final manuscript.
